# Different mol­ecular conformations in the crystal structures of three 5-nitro­imidazolyl derivatives

**DOI:** 10.1107/S2056989018002876

**Published:** 2018-02-23

**Authors:** Luis F. B. Osorio, Samir A. Carvalho, Edson F. da Silva, Carlos A. M. Fraga, Solange M. S. V. Wardell, Bruce F. Milne, James L. Wardell, William T. A. Harrison

**Affiliations:** a Instituto de Tecnologia em Fármacos e Farmanguinhos, Fundação Oswaldo Cruz, 21041-250 Rio de Janeiro, RJ, Brazil; b Programa de Pesquisa em Desenvolvimento de Fármacos, Instituto de Ciências Biomédicas, Universidade Federal do Rio de Janeiro, PO Box 68023, 21941-902 Rio de Janeiro, RJ, Brazil; c CHEMSOL, 1 Harcourt Road, Aberdeen AB15 5NY, Scotland; d CFisUC, Physics Department, University of Coimbra, Rua Larga 3004–516, Coimbra, Portugal; e Department of Chemistry, University of Aberdeen, Meston Walk, Aberdeen AB24 3UE, Scotland

**Keywords:** benzoxa­thiol-2-one, hydrogen bonds, Hirshfeld surface, crystal structure

## Abstract

The crystal structures of (*E*)-1-methyl-5-nitro-1*H*-imidazole-2-carbaldehyde *O*-benzyl­oxime, C_12_H_12_N_4_O_3_, (I), (*E*)-1-methyl-5-nitro-1*H*-imidazole-2-carb­alde­hyde *O*-(4-fluoro­benz­yl) oxime, C_12_H_11_FN_4_O_3_, (II), and (*E*)-1-methyl-5-nitro-1*H*-imidazole-2-carbaldehyde *O*-(4-bromo­benz­yl) oxime, C_12_H_11_BrN_4_O_3_, (III), are described. The dihedral angle between the ring systems in (I) is 49.66 (5)° and the linking N_m_—C—C=N (m = methyl­ated) bond shows an *anti* conformation [torsion angle = 175.00 (15)°]. Compounds (II) and (III) are isostructural [dihedral angle between the aromatic rings = 8.31 (5)° in (II) and 5.34 (15)° in (III)] and differ from (I) in showing a near-*syn* conformation for the N_m_—C—C=N linker [torsion angles for (II) and (III) = 17.64 (18) and 8.7 (5)°, respectively], which allows for the occurrence of a short intra­molecular C—H⋯N contact. In the crystal of (I), C—H⋯N hydrogen bonds link the mol­ecules into [010] chains, which are cross-linked by very weak C—H⋯O bonds into (100) sheets. Weak aromatic π–π stacking inter­actions occur between the sheets. The extended structures of (II) and (III) feature several C—H⋯N and C—H⋯O hydrogen bonds, which link the mol­ecules into three-dimensional networks, which are consolidated by aromatic π–π stacking inter­actions. Conformational energy calculations and Hirshfeld fingerprint analyses for (I), (II) and (III) are presented and discussed.

## Abstract 2

The title compounds show different conformations in the solid state.

## Chemical context   

Trypanosomes infect a variety of hosts and cause various serious illnesses, including sleeping sickness (transmitted by *Trypanosoma brucei*) and Chagas’ disease. The infectious agent of Chagas’ disease is the protozoan parasite *Trypanosoma cruzi*, which produces progressive symptoms from mild swelling to intestinal disease and ultimately heart failure (Rassi *et al.*, 2010 ▸). New effective drugs are urgently required for the treatment of Chagas’ disease, which infects an estimated 6.6 million people worldwide (Rassi *et al.*, 2010 ▸): benznidazole and nifurtimox have been the only recognised treatments for over 40 years and both drugs present variable results and undesirable side effects (Soeiro & Castro, 2011 ▸). Megazol, while active, also has serious side effects (Poli *et al.* 2002 ▸).

We have recently described (Carvalho *et al.*, 2017 ▸) the syntheses and biological activities of a family of 5-nitro­imidazolyl-*O*-benzyl­oxime ethers, which displayed moderate anti­trypanosidal activity. We now report the crystal structures, Hirshfeld surface analyses and conformational energy calculations for three compounds from that study, *viz*. (*E*)-1-methyl-5-nitro-1*H*-imidazole-2-carbaldehyde *O*-benzyl­oxime, C_12_H_12_N_4_O_3_ (I), (*E*)-1-methyl-5-nitro-1*H*-imidazole-2-carb­al­de­hyde *O*-(4-fluoro­benz­yl) oxime C_12_H_11_FN_4_O_3_ (II) and (*E*)-1-methyl-5-nitro-1*H*-imidazole-2-carbaldehyde *O*-(4-bromo­benz­yl) oxime, C_12_H_11_BrN_4_O_3_ (III).
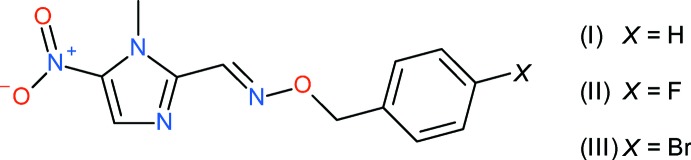


## Structural commentary   

**Figure 1:**
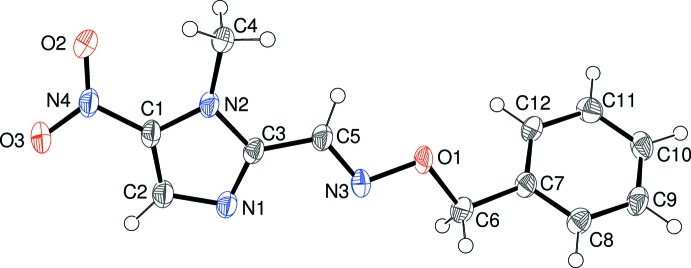
The mol­ecular structure of (I) showing 50% displacement ellipsoids.

Compound (I) crystallizes in space group *P*2_1_/*c* with one mol­ecule in the asymmetric unit (Fig. 1 and Table 1 ▸ ▸). The dihedral angle between the imidazole ring (C1/C2/C3/N1/N2) and phenyl group (C7–C12) is 49.66 (5)°. The N4/O2/O3 nitro group is approximately coplanar with its attached ring [dihedral angle = 7.87 (17)°]. The C—C and C—N bond lengths within the heterocyclic ring show typical values and N2 is statistically planar (bond-angle sum = 359.7°). The angle C1—N2—C4 [129.47 (13)°] is significantly greater than C3—N2—C4 [126.14 (14)°] perhaps because of steric repulsion between the C4 methyl group and the nitro group. The key parameter defining the conformation of the mol­ecule of (I) is the N2—C3—C5=N3 torsion angle: the value of 175.00 (15)° indicates an *anti* conformation for these atoms. The rest of the chain linking the rings can be described as extended in terms of the C3—C5=N3—O1, C5=N3—O1—C6 and N3—O1—C6—C7 torsion angles of 175.55 (14), −172.50 (15) and 172.62 (14)°, respectively. The major twist in the mol­ecule of (I) occurs about the C6—C7 bond as indicated by the O1—C6—C7—C12 torsion angle of −45.5 (2)°. Assuming that the rotating-group refinement model for the C4 methyl group is reliable, it may be seen that this group has twisted about the N2—C4 bond to reduce steric repulsion with H5, although a rather short intra­molecular contact (H5⋯H4*C* = 2.12 Å) is still present.

**Figure 2:**
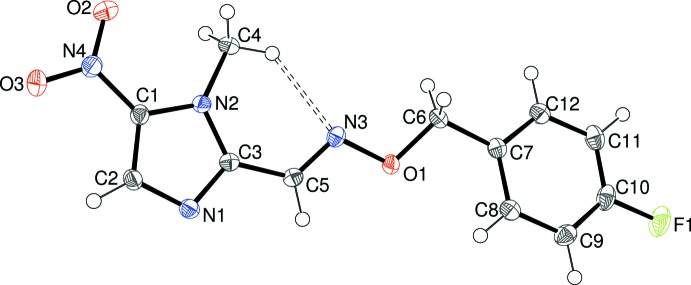
The mol­ecular structure of (II) showing 50% displacement ellipsoids.

Compounds (II) and (III) are isostructural, crystallizing in *P*2_1_/*n* with one mol­ecule in the asymmetric unit (Figs. 2 ▸ and 3 ▸). The dihedral angles between the aromatic rings for (II) and (III) are 8.31 (5) and 5.34 (15)°, respectively, whereas the dihedral angles for the nitro group and its attached ring are 2.83 (11) and 5.9 (30)°, respectively. The geometrical data for the imidazole rings in (II) and (III) show no significant differences compared to (I) but a major conformational difference is seen in terms of the N2—C3—C5=N3 torsion angles of 17.64 (18) for (II) and 8.7 (5)° for (III), indicating an approximate *syn* conformation, as opposed to *anti* for (I). This reorientation facilitates the formation of an intra­molecular C4—H4*C*⋯N3 hydrogen bond in both (II) (Table 2 ▸) and (III) (Table 3 ▸). The rest of the linking chain displays an extended conformation in both (II) and (III) with respective C3—C5=N3—O1, C5=N3—O1—C6 and N3—O1—C6—C7 torsion angles of 179.79 (9), −173.96 (9) and 175.61 (8)° in (II) and 179.2 (2), −171.8 (2) and 179.7 (2)° in (III). The C6—C7 bond in (II) and (III) is somewhat less twisted than in (I), with O1—C6—C7—C8 torsion angles of −30.95 (14) and −23.1 (4)° for (II) and (III), respectively.

## Computational calculations   

The different conformations of (I) compared to (II) and (III) were investigated by computational means. All calculations were performed with the *Orca* software package version 4.0.0.2 (Neese, 2012 ▸). Geometry optimizations were performed at the spin-component-scaled MP2 (SCS-MP2) level (Grimme, 2003 ▸) using the Def2-TZVP (Hellweg *et al.*, 2007 ▸) basis set. Optimized geometries were then subjected to single-point energy calculations at the SCS-MP2 level with the larger Def2-QZVPP basis set to obtain final relative conformational energies. Geometry optimizations and single point energies were repeated using the SMD method to model the methanol solvent environment (Marenich *et al.*, 2009 ▸) used in the crystallization experiments. The results (Table 4 ▸) show that the *syn* conformation [*i.e*. that found for (II) and (III)] is favoured for all substituents by roughly the same energy (with the energy of the *syn* conformer arbitrarily defined to be zero in each case) either *in* vacuo or in a methanol solvent environment, although the differences in the latter case are quite small.

## Supra­molecular features   

**Figure 4:**
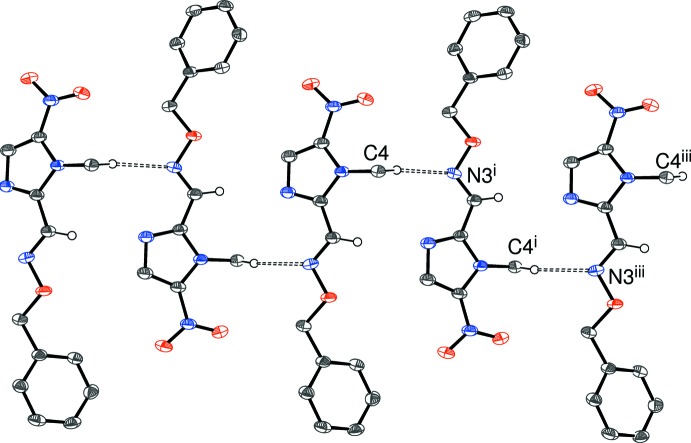
Fragment of an [010] hydrogen-bonded chain in the crystal of (I). Symmetry codes: (i) –x, 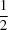 + *y*, 1/2 – z; (iii) *x*, *y* + 1, *z*.

In the crystal of (I), the mol­ecules are linked by C—H⋯N hydrogen bonds (Table 1 ▸) to generate [010] *C*(6) chains, with adjacent mol­ecules related by the 2_1_ screw axis (Fig. 4 ▸). The C5—H5⋯O3 contact is long and the angle is small, but if it is regarded as significant, it serves to cross-link the chains into (100) sheets. Weak aromatic π–π stacking inter­actions arise between the sheets, such that each imidazole ring is sandwiched by two phenyl groups and *vice versa* [centroid–centroid separations = 3.7355 (10) and 4.1184 (10) Å; corres­ponding slippages = 1.35 and 2.25 Å, respectively].

There are a number of inter­molecular inter­actions in (II) (Table 2 ▸) and (III) (Table 3 ▸) and together they lead to three-dimensional networks in each case. It is inter­esting that the C9—H9⋯N1 inter­action in (II) is clearly a directional bond [H⋯N = 2.58 Å compared to a van der Waals contact distance (Bondi, 1964 ▸) of 2.75 Å for these atoms] whereas the equivalent contact in (III), included in Table 3 ▸ for completeness, has an H⋯N separation of 2.77 Å and, by itself, would be very doubtful as a bond, which shows that isostructural crystals can show distinct variations in their weak inter­actions. This is supported by the presence of a weak C4—H4*C*⋯Br1 bond in (III) (H⋯Br = 2.85 Å, van der Waals contact distance = 3.05 Å) whilst the equivalent link in (II) has H⋯F = 2.77 Å, significantly greater than the van der Waals contact distance of 2.67 Å and would not be regarded as a significant bond. As in (I), π–π stacking appears to consolidate the crystals of (II) and (III), in which the imidazole rings and phenyl rings form alternating stacks, which propagate in [100]. In (II), the imidazole ring faces phenyl rings with centroid–centroid (slippage) distances of 3.7297 (7) (1.23) and 3.9323 (7) Å (1.64 Å). Equivalent data for (III) are 3.7664 (18) (1.47) and 3.9698 (18) Å (1.82 Å).

## Hirshfeld surface analysis   

Hirshfeld surface fingerprint plots for (I), (II) and (III) (supplementary Figs. 1 ▸, 2 ▸ and 3 ▸, respectively) were calculated with *CrystalExplorer17* (Turner *et al.*, 2017 ▸). When the fingerprint plots are decomposed into the separate types of inter­molecular contacts (McKinnon *et al.*, 2007 ▸), it may be seen (Table 5 ▸) that as a percentage of surface inter­actions, H⋯H contacts (*i.e*. van der Waals inter­actions) are the most significant in each structure, followed by O⋯H/H⋯O contacts. It is inter­esting the percentage of the latter for (I) is slightly higher than for (II), despite the fact that (I) features one weak C—H⋯O bond at best whilst (II) features three such bonds. The C⋯C contacts (associated with aromatic π–π stacking) contribute a very small percentage in each structure, which is slightly surprising given the significant π–π stacking inter­actions noted above. Finally, it may be noted that the C⋯H/H⋯C and N⋯N/H⋯N contributions for (I) and the C⋯H/H⋯C, N⋯N/H⋯N and *X*—H/H⋯*X* contributions for (II) and (III) sum to approximately the same amount.

Beyond a vague appeal to ‘packing forces’, we find it difficult to explain why (I) forms the energetically disfavoured *anti* conformation in the crystal: it allows the C5—H5 group to form a weak hydrogen bond (Table 1 ▸) to a nitro group oxygen atom but it should be noted that the same grouping forms a similar bond in the opposite direction (*i.e*. pointing away from C4) in both (II) and (III). The *syn* conformation for (II) and (III) seems to be favoured in terms of the occurrence of an intra­molecular C—H⋯N link and it is possible that weak C—H⋯*X* (*X* = F, Br) inter­actions in the crystals of (II) and (III) provide some stabilization not possible in (I), although they are at the opposite end of the mol­ecule. The Hirshfeld fingerprint data (Table 5 ▸) show that N⋯H/H⋯N and C⋯H/H⋯C contacts are somewhat more significant in the crystal of (I) but the energetic consequences of these are not clear. We cannot rule out the posssibility that a polymorph of (I) may exist in which the N_m_—C—C=N grouping has a *syn* conformation but with a different overall packing motif to (II) and (III).

## Database survey   

A survey of of the Cambridge Structural Database (Groom *et al.*, 2016 ▸: updated to January 2018) for the 1-methyl 5-nitro imidazole fragment revealed 33 hits. The 4-methyl-substituted analogue of the title compounds, *N*-[(4-methyl­benz­yl)­oxy]-1-(1-methyl-5-nitro-1*H*-imidazol-2-yl)methanimine (refcode: TEVGAF), has been reported by Carvalho *et al.* (2017 ▸): its N_m_—C—C=N torsion angle is −30.7 (2)°, *i.e*. somewhat twisted from *syn*.

## Synthesis and crystallization   

The syntheses and spectroscopic data of the title compounds have already been described (Carvalho *et al.*, 2017 ▸). The crystals used for data collections in this study were recrystallized from methanol solution in each case as colourless plates of (I), orange blocks of (II) and yellow blocks of (III).

## Refinement   

Crystal data, data collection and structure refinement details are summarized in Table 6 ▸. The hydrogen atoms were geometrically placed (C—H = 0.95–0.99Å) and refined as riding atoms. The constraint *U*_iso_(H) = 1.2*U*_eq_(carrier) or 1.5*U*_eq_(methyl carrier) was applied in all cases. The methyl groups were allowed to rotate, but not to tip, to best fit the electron density.

## Supplementary Material

Crystal structure: contains datablock(s) I, II, III, global. DOI: 10.1107/S2056989018002876/mw2136sup1.cif

Structure factors: contains datablock(s) I. DOI: 10.1107/S2056989018002876/mw2136Isup2.hkl

Supporting information file. DOI: 10.1107/S2056989018002876/mw2136Isup5.cml

Structure factors: contains datablock(s) II. DOI: 10.1107/S2056989018002876/mw2136IIsup3.hkl

Supporting information file. DOI: 10.1107/S2056989018002876/mw2136IIsup6.cml

Structure factors: contains datablock(s) III. DOI: 10.1107/S2056989018002876/mw2136IIIsup4.hkl

Supporting information file. DOI: 10.1107/S2056989018002876/mw2136IIIsup7.cml

Supplementary Figures 1, 2 and 3: Hirshfeld fingerprint plots. DOI: 10.1107/S2056989018002876/mw2136sup8.pdf

CCDC references: 1486983, 1486982, 1486987

Additional supporting information:  crystallographic information; 3D view; checkCIF report

## Figures and Tables

**Figure 3:**
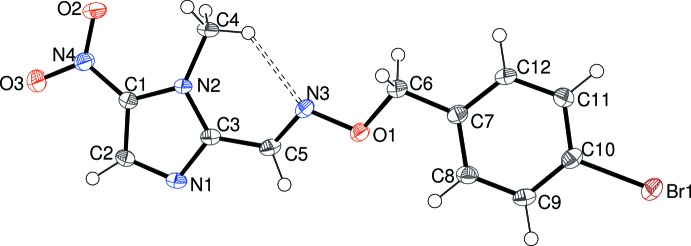
The mol­ecular structure of (III) showing 50% displacement ellipsoids.

**Table 1:** Hydrogen-bond geometry (Å, °) for (I)

*D*—H⋯*A*	*D*—H	H⋯*A*	*D*⋯*A*	*D*—H⋯*A*
C4—H4*B*⋯N3^i^	0.98	2.51	3.466 (2)	165
C5—H5⋯O3^ii^	0.95	2.65	3.175 (2)	115

Symmetry codes: (i) 
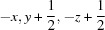
; (ii) 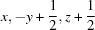.

**Table 2:** Hydrogen-bond geometry (Å, °) for (II)

*D*—H⋯*A*	*D*—H	H⋯*A*	*D*⋯*A*	*D*—H⋯*A*
C4—H4*C*⋯N3	0.98	2.29	3.0184 (15)	131
C4—H4*A*⋯N1^i^	0.98	2.63	3.5693 (16)	160
C9—H9⋯N1^ii^	0.95	2.58	3.4973 (16)	163
C2—H2⋯O3^iii^	0.95	2.49	3.3165 (15)	145
C5—H5⋯O2^iv^	0.95	2.63	3.1676 (14)	116
C6—H6*A*⋯O2^v^	0.99	2.54	3.1376 (14)	119
C4—H4*C*⋯F1^vi^	0.98	2.77	3.353 (2)	119

Symmetry codes: (i) 
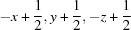
; (ii) 
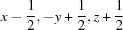
; (iii) 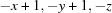; (iv) 
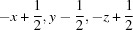
; (v) 
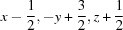
; (vi) 
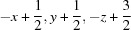
.

**Table 3:** Hydrogen-bond geometry (Å, °) for (III)

*D*—H⋯*A*	*D*—H	H⋯*A*	*D*⋯*A*	*D*—H⋯*A*
C4—H4*C*⋯N3	0.98	2.24	2.997 (4)	133
C4—H4*A*⋯N1^i^	0.98	2.62	3.499 (4)	149
C9—H9⋯N1^ii^	0.95	2.77	3.681 (4)	160
C2—H2⋯O3^iii^	0.95	2.44	3.282 (4)	148
C5—H5⋯O2^iv^	0.95	2.64	3.341 (4)	131
C6—H6*A*⋯O2^v^	0.99	2.63	3.254 (4)	121
C4—H4*C*⋯Br1^vi^	0.98	2.85	3.491 (3)	124

Symmetry codes: (i) 
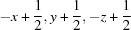
; (ii) 
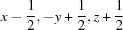
; (iii) 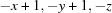; (iv) 
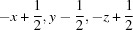
; (v) 
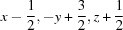
; (vi) 
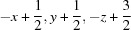
.

**Table 4:** Relative conformational energies (kJ mol^−1^) The two values refer to a vacuum and methanol solvation, respectively. The energy of the *syn* conformer is arbitrarily set to zero in each case.

Substituent	Compound	*anti*	*syn*
H	(I)	14.90/5.91	0
CH_3_	Carvalho *et al.* (2017 ▸)	14.90/6.84	0
F	(II)	17.12/6.17	0
Br	(III)	16.84/6.17	0

**Table 5:** Hirshfeld contact inter­actions (%)

Contact type	(I)	(II)	(III)
H⋯H	34.6	30.3	28.3
O⋯H/H⋯O	24.6	24.4	23.2
N⋯H/H⋯N	14.7	9.4	8.1
C⋯H/H⋯C	12.4	6.0	6.5
C⋯C	4.6	5.8	5.9
*X*⋯H/H⋯*X*	–	11.7	15.0

**Table 6:** Experimental details

	(I)	(II)	(III)
Crystal data			
Chemical formula	C_12_H_12_N_4_O_3_	C_12_H_11_FN_4_O_3_	C_12_H_11_BrN_4_O_3_
*M*_r_	260.26	278.25	339.16
Crystal system, space group	Monoclinic, *P*2_1_/*c*	Monoclinic, *P*2_1_/*n*	Monoclinic, *P*2_1_/*n*
Temperature (K)	100	120	120
*a*, *b*, *c* (Å)	7.6399 (5), 10.5071 (7), 14.9243 (11)	7.5484 (2), 12.6442 (4), 13.4150 (9)	7.6024 (2), 12.7526 (3), 13.8954 (5)
β (°)	97.942 (3)	102.988 (7)	104.869 (2)
*V* (Å^3^)	1186.53 (14)	1247.62 (10)	1302.05 (7)
*Z*	4	4	4
Radiation type	Mo *K*α	Mo *K*α	Mo *K*α
μ (mm^−1^)	0.11	0.12	3.17
Crystal size (mm)	0.11 × 0.07 × 0.03	0.19 × 0.13 × 0.10	0.66 × 0.52 × 0.24

Data collection			
Diffractometer	Rigaku Saturn724+ CCD	Rigaku Saturn724+ CCD	Rigaku Mercury CCD
Absorption correction	Multi-scan (*FS_ABSCOR*; Rigaku, 2013 ▸)	Multi-scan (*FS_ABSCOR*; Rigaku, 2013 ▸)	Multi-scan (*FS_ABSCOR*; Rigaku, 2013 ▸)
*T*_min_, *T*_max_	0.618, 1.000	0.802, 1.000	0.438, 1.000
No. of measured, independent and observed [*I* > 2σ(*I*)] reflections	7757, 2705, 1891	8522, 2848, 2292	16791, 2974, 2835
*R*_int_	0.055	0.021	0.065
(sin θ/λ)_max_ (Å^−1^)	0.649	0.649	0.650

Refinement			
*R*[*F* ^2^ > 2σ(*F* ^2^)], *wR*(*F* ^2^), *S*	0.047, 0.126, 0.95	0.034, 0.094, 1.09	0.051, 0.140, 1.11
No. of reflections	2705	2848	2974
No. of parameters	173	182	182
H-atom treatment	H-atom parameters constrained	H-atom parameters constrained	H-atom parameters constrained
Δρ_max_, Δρ_min_ (e Å^−3^)	0.24, −0.22	0.29, −0.18	1.78, −1.03

Computer programs: *CrystalClear* (Rigaku, 2012 ▸), *SHELXS97* (Sheldrick, 2008 ▸), *SHELXL2014* (Sheldrick, 2015 ▸), *ORTEP-3 for Windows* (Farrugia, 2012 ▸) and *publCIF* (Westrip, 2010 ▸).

## supplementary crystallographic information

### (E)-1-Methyl-5-nitro-1H-imidazole-2-carbaldehyde O-benzyloxime (I) Crystal data

**Table:**

C_12_H_12_N_4_O_3_	*F*(000) = 544
*M**_r_* = 260.26	*D*_x_ = 1.457 Mg m^−^^3^
Monoclinic, *P*2_1_/*c*	Mo *K*α radiation, λ = 0.71073 Å
*a* = 7.6399 (5) Å	Cell parameters from 7971 reflections
*b* = 10.5071 (7) Å	θ = 2.4–27.5°
*c* = 14.9243 (11) Å	µ = 0.11 mm^−^^1^
β = 97.942 (3)°	*T* = 100 K
*V* = 1186.53 (14) Å^3^	Plate, colourless
*Z* = 4	0.11 × 0.07 × 0.03 mm

### (E)-1-Methyl-5-nitro-1H-imidazole-2-carbaldehyde O-benzyloxime (I) Data collection

**Table:**

Rigaku Saturn724+ CCD diffractometer	1891 reflections with *I* > 2σ(*I*)
ω scans	*R*_int_ = 0.055
Absorption correction: multi-scan (*FS_ABSCOR*; Rigaku, 2013)	θ_max_ = 27.5°, θ_min_ = 2.4°
*T*_min_ = 0.618, *T*_max_ = 1.000	*h* = −9→6
7757 measured reflections	*k* = −13→13
2705 independent reflections	*l* = −19→18

### (E)-1-Methyl-5-nitro-1H-imidazole-2-carbaldehyde O-benzyloxime (I) Refinement

**Table:**

Refinement on *F*^2^	Primary atom site location: structure-invariant direct methods
Least-squares matrix: full	Hydrogen site location: inferred from neighbouring sites
*R*[*F*^2^ > 2σ(*F*^2^)] = 0.047	H-atom parameters constrained
*wR*(*F*^2^) = 0.126	*w* = 1/[σ^2^(*F*_o_^2^) + (0.0674*P*)^2^] where *P* = (*F*_o_^2^ + 2*F*_c_^2^)/3
*S* = 0.95	(Δ/σ)_max_ < 0.001
2705 reflections	Δρ_max_ = 0.24 e Å^−^^3^
173 parameters	Δρ_min_ = −0.22 e Å^−^^3^
0 restraints	

### (E)-1-Methyl-5-nitro-1H-imidazole-2-carbaldehyde O-benzyloxime (I) Special details

**Table:**

Geometry. All esds (except the esd in the dihedral angle between two l.s. planes) are estimated using the full covariance matrix. The cell esds are taken into account individually in the estimation of esds in distances, angles and torsion angles; correlations between esds in cell parameters are only used when they are defined by crystal symmetry. An approximate (isotropic) treatment of cell esds is used for estimating esds involving l.s. planes.

### (E)-1-Methyl-5-nitro-1H-imidazole-2-carbaldehyde O-benzyloxime (I) Fractional atomic coordinates and isotropic or equivalent isotropic displacement parameters (Å^2^)

**Table:**

	*x*	*y*	*z*	*U*_iso_*/*U*_eq_	
C1	−0.2026 (2)	0.18424 (17)	0.08287 (11)	0.0272 (4)	
C2	−0.1242 (2)	0.06989 (17)	0.10516 (11)	0.0302 (4)	
H2	−0.1177	0.0010	0.0646	0.036*	
C3	−0.0953 (2)	0.18453 (17)	0.22478 (11)	0.0262 (4)	
C4	−0.2593 (2)	0.38590 (16)	0.17211 (11)	0.0295 (4)	
H4A	−0.3780	0.3918	0.1378	0.044*	
H4B	−0.1832	0.4512	0.1508	0.044*	
H4C	−0.2661	0.3991	0.2365	0.044*	
C5	−0.0422 (2)	0.23260 (16)	0.31595 (11)	0.0277 (4)	
H5	−0.0610	0.3193	0.3300	0.033*	
C6	0.1846 (3)	0.13647 (17)	0.51983 (12)	0.0353 (5)	
H6A	0.2794	0.0969	0.4903	0.042*	
H6B	0.1069	0.0680	0.5372	0.042*	
C7	0.2641 (2)	0.20824 (16)	0.60261 (11)	0.0275 (4)	
C8	0.2637 (2)	0.15343 (17)	0.68727 (11)	0.0296 (4)	
H8	0.2039	0.0751	0.6927	0.036*	
C9	0.3508 (2)	0.21295 (18)	0.76444 (12)	0.0328 (4)	
H9	0.3522	0.1739	0.8220	0.039*	
C10	0.4346 (2)	0.32796 (18)	0.75770 (12)	0.0336 (4)	
H10	0.4943	0.3680	0.8103	0.040*	
C11	0.4313 (2)	0.38490 (17)	0.67342 (12)	0.0327 (4)	
H11	0.4865	0.4652	0.6686	0.039*	
C12	0.3475 (2)	0.32503 (17)	0.59602 (11)	0.0293 (4)	
H12	0.3471	0.3639	0.5385	0.035*	
N1	−0.05705 (18)	0.06958 (14)	0.19410 (9)	0.0292 (4)	
N2	−0.18520 (17)	0.25946 (13)	0.15896 (9)	0.0257 (3)	
N3	0.03028 (18)	0.15619 (14)	0.37682 (9)	0.0287 (3)	
N4	−0.28663 (19)	0.22404 (14)	−0.00435 (9)	0.0308 (4)	
O1	0.08352 (16)	0.22291 (11)	0.45775 (7)	0.0304 (3)	
O2	−0.33264 (17)	0.33549 (12)	−0.01644 (8)	0.0382 (3)	
O3	−0.30669 (18)	0.14106 (13)	−0.06357 (8)	0.0402 (4)	

### (E)-1-Methyl-5-nitro-1H-imidazole-2-carbaldehyde O-benzyloxime (I) Atomic displacement parameters (Å^2^)

**Table:**

	*U*^11^	*U*^22^	*U*^33^	*U*^12^	*U*^13^	*U*^23^
C1	0.0262 (8)	0.0346 (9)	0.0192 (8)	−0.0040 (7)	−0.0031 (6)	0.0003 (7)
C2	0.0309 (9)	0.0354 (9)	0.0224 (9)	−0.0024 (8)	−0.0026 (7)	−0.0007 (7)
C3	0.0231 (8)	0.0319 (9)	0.0215 (8)	−0.0024 (7)	−0.0038 (6)	0.0032 (7)
C4	0.0303 (9)	0.0299 (8)	0.0257 (9)	0.0015 (7)	−0.0055 (7)	0.0003 (7)
C5	0.0276 (9)	0.0319 (8)	0.0215 (8)	−0.0012 (7)	−0.0033 (7)	0.0000 (7)
C6	0.0412 (10)	0.0328 (9)	0.0270 (9)	0.0019 (8)	−0.0131 (8)	0.0024 (8)
C7	0.0243 (8)	0.0320 (9)	0.0232 (8)	0.0031 (7)	−0.0070 (6)	0.0001 (7)
C8	0.0266 (8)	0.0335 (9)	0.0272 (9)	0.0006 (7)	−0.0018 (7)	0.0034 (7)
C9	0.0328 (9)	0.0427 (10)	0.0211 (9)	0.0104 (8)	−0.0030 (7)	0.0024 (8)
C10	0.0305 (9)	0.0404 (10)	0.0263 (9)	0.0069 (8)	−0.0090 (7)	−0.0072 (8)
C11	0.0271 (9)	0.0317 (9)	0.0366 (10)	−0.0005 (7)	−0.0049 (7)	−0.0031 (8)
C12	0.0285 (9)	0.0344 (9)	0.0230 (9)	0.0024 (8)	−0.0031 (7)	0.0037 (7)
N1	0.0306 (7)	0.0339 (8)	0.0212 (7)	−0.0019 (6)	−0.0033 (6)	0.0012 (6)
N2	0.0247 (7)	0.0305 (7)	0.0197 (7)	−0.0012 (6)	−0.0049 (6)	0.0002 (6)
N3	0.0282 (7)	0.0355 (8)	0.0203 (7)	−0.0027 (6)	−0.0040 (6)	−0.0042 (6)
N4	0.0321 (8)	0.0374 (8)	0.0202 (7)	−0.0036 (7)	−0.0054 (6)	0.0002 (7)
O1	0.0334 (7)	0.0358 (6)	0.0185 (6)	0.0028 (5)	−0.0087 (5)	−0.0025 (5)
O2	0.0460 (8)	0.0378 (7)	0.0271 (7)	0.0049 (6)	−0.0085 (6)	0.0048 (6)
O3	0.0500 (8)	0.0428 (7)	0.0236 (7)	−0.0058 (6)	−0.0097 (6)	−0.0054 (6)

### (E)-1-Methyl-5-nitro-1H-imidazole-2-carbaldehyde O-benzyloxime (I) Geometric parameters (Å, º)

**Table:**

C1—C2	1.363 (2)	C6—H6A	0.9900
C1—N2	1.375 (2)	C6—H6B	0.9900
C1—N4	1.432 (2)	C7—C8	1.389 (2)
C2—N1	1.355 (2)	C7—C12	1.392 (2)
C2—H2	0.9500	C8—C9	1.396 (2)
C3—N1	1.338 (2)	C8—H8	0.9500
C3—N2	1.368 (2)	C9—C10	1.378 (3)
C3—C5	1.456 (2)	C9—H9	0.9500
C4—N2	1.468 (2)	C10—C11	1.390 (3)
C4—H4A	0.9800	C10—H10	0.9500
C4—H4B	0.9800	C11—C12	1.391 (2)
C4—H4C	0.9800	C11—H11	0.9500
C5—N3	1.279 (2)	C12—H12	0.9500
C5—H5	0.9500	N3—O1	1.4071 (16)
C6—O1	1.4428 (19)	N4—O2	1.2287 (18)
C6—C7	1.502 (2)	N4—O3	1.2359 (18)
			
C2—C1—N2	108.43 (14)	C12—C7—C6	121.42 (15)
C2—C1—N4	127.30 (16)	C7—C8—C9	120.27 (17)
N2—C1—N4	124.26 (15)	C7—C8—H8	119.9
N1—C2—C1	109.59 (15)	C9—C8—H8	119.9
N1—C2—H2	125.2	C10—C9—C8	120.40 (16)
C1—C2—H2	125.2	C10—C9—H9	119.8
N1—C3—N2	112.68 (14)	C8—C9—H9	119.8
N1—C3—C5	125.95 (14)	C9—C10—C11	119.54 (16)
N2—C3—C5	121.28 (15)	C9—C10—H10	120.2
N2—C4—H4A	109.5	C11—C10—H10	120.2
N2—C4—H4B	109.5	C10—C11—C12	120.36 (17)
H4A—C4—H4B	109.5	C10—C11—H11	119.8
N2—C4—H4C	109.5	C12—C11—H11	119.8
H4A—C4—H4C	109.5	C11—C12—C7	120.16 (16)
H4B—C4—H4C	109.5	C11—C12—H12	119.9
N3—C5—C3	118.90 (15)	C7—C12—H12	119.9
N3—C5—H5	120.5	C3—N1—C2	105.18 (14)
C3—C5—H5	120.5	C3—N2—C1	104.12 (14)
O1—C6—C7	109.39 (14)	C3—N2—C4	126.14 (14)
O1—C6—H6A	109.8	C1—N2—C4	129.47 (13)
C7—C6—H6A	109.8	C5—N3—O1	110.01 (13)
O1—C6—H6B	109.8	O2—N4—O3	124.23 (14)
C7—C6—H6B	109.8	O2—N4—C1	119.68 (14)
H6A—C6—H6B	108.2	O3—N4—C1	116.09 (14)
C8—C7—C12	119.23 (15)	N3—O1—C6	107.65 (12)
C8—C7—C6	119.24 (16)		
			
N2—C1—C2—N1	0.2 (2)	C1—C2—N1—C3	−0.16 (19)
N4—C1—C2—N1	178.92 (15)	N1—C3—N2—C1	0.01 (19)
N1—C3—C5—N3	−8.6 (3)	C5—C3—N2—C1	176.86 (15)
N2—C3—C5—N3	175.00 (15)	N1—C3—N2—C4	174.49 (15)
O1—C6—C7—C8	138.31 (16)	C5—C3—N2—C4	−8.7 (2)
O1—C6—C7—C12	−45.5 (2)	C2—C1—N2—C3	−0.11 (18)
C12—C7—C8—C9	−2.0 (3)	N4—C1—N2—C3	−178.90 (15)
C6—C7—C8—C9	174.21 (16)	C2—C1—N2—C4	−174.33 (16)
C7—C8—C9—C10	1.4 (3)	N4—C1—N2—C4	6.9 (3)
C8—C9—C10—C11	0.3 (3)	C3—C5—N3—O1	175.55 (14)
C9—C10—C11—C12	−1.5 (3)	C2—C1—N4—O2	−171.21 (18)
C10—C11—C12—C7	0.9 (3)	N2—C1—N4—O2	7.4 (3)
C8—C7—C12—C11	0.9 (3)	C2—C1—N4—O3	8.3 (3)
C6—C7—C12—C11	−175.28 (16)	N2—C1—N4—O3	−173.14 (16)
N2—C3—N1—C2	0.09 (19)	C5—N3—O1—C6	−172.50 (15)
C5—C3—N1—C2	−176.59 (16)	C7—C6—O1—N3	172.62 (14)

### (E)-1-Methyl-5-nitro-1H-imidazole-2-carbaldehyde O-benzyloxime (I) Hydrogen-bond geometry (Å, º)

**Table:**

*D*—H···*A*	*D*—H	H···*A*	*D*···*A*	*D*—H···*A*
C4—H4*B*···N3^i^	0.98	2.51	3.466 (2)	165
C5—H5···O3^ii^	0.95	2.65	3.175 (2)	115

Symmetry codes: (i) −*x*, *y*+1/2, −*z*+1/2; (ii) *x*, −*y*+1/2, *z*+1/2.

### (E)-1-Methyl-5-nitro-1H-imidazole-2-carbaldehyde O-(4-fluorobenzyl) oxime (II) Crystal data

**Table:**

C_12_H_11_FN_4_O_3_	*F*(000) = 576
*M**_r_* = 278.25	*D*_x_ = 1.481 Mg m^−^^3^
Monoclinic, *P*2_1_/*n*	Mo *K*α radiation, λ = 0.71073 Å
*a* = 7.5484 (2) Å	Cell parameters from 7472 reflections
*b* = 12.6442 (4) Å	θ = 3.1–27.5°
*c* = 13.4150 (9) Å	µ = 0.12 mm^−^^1^
β = 102.988 (7)°	*T* = 120 K
*V* = 1247.62 (10) Å^3^	Block, orange
*Z* = 4	0.19 × 0.13 × 0.10 mm

### (E)-1-Methyl-5-nitro-1H-imidazole-2-carbaldehyde O-(4-fluorobenzyl) oxime (II) Data collection

**Table:**

Rigaku Saturn724+ CCD diffractometer	2292 reflections with *I* > 2σ(*I*)
ω scans	*R*_int_ = 0.021
Absorption correction: multi-scan (*FS_ABSCOR*; Rigaku, 2013)	θ_max_ = 27.5°, θ_min_ = 3.1°
*T*_min_ = 0.802, *T*_max_ = 1.000	*h* = −9→9
8522 measured reflections	*k* = −14→16
2848 independent reflections	*l* = −14→17

### (E)-1-Methyl-5-nitro-1H-imidazole-2-carbaldehyde O-(4-fluorobenzyl) oxime (II) Refinement

**Table:**

Refinement on *F*^2^	Primary atom site location: structure-invariant direct methods
Least-squares matrix: full	Hydrogen site location: inferred from neighbouring sites
*R*[*F*^2^ > 2σ(*F*^2^)] = 0.034	H-atom parameters constrained
*wR*(*F*^2^) = 0.094	*w* = 1/[σ^2^(*F*_o_^2^) + (0.0501*P*)^2^ + 0.1808*P*] where *P* = (*F*_o_^2^ + 2*F*_c_^2^)/3
*S* = 1.09	(Δ/σ)_max_ < 0.001
2848 reflections	Δρ_max_ = 0.29 e Å^−^^3^
182 parameters	Δρ_min_ = −0.18 e Å^−^^3^
0 restraints	

### (E)-1-Methyl-5-nitro-1H-imidazole-2-carbaldehyde O-(4-fluorobenzyl) oxime (II) Special details

**Table:**

Geometry. All esds (except the esd in the dihedral angle between two l.s. planes) are estimated using the full covariance matrix. The cell esds are taken into account individually in the estimation of esds in distances, angles and torsion angles; correlations between esds in cell parameters are only used when they are defined by crystal symmetry. An approximate (isotropic) treatment of cell esds is used for estimating esds involving l.s. planes.

### (E)-1-Methyl-5-nitro-1H-imidazole-2-carbaldehyde O-(4-fluorobenzyl) oxime (II) Fractional atomic coordinates and isotropic or equivalent isotropic displacement parameters (Å^2^)

**Table:**

	*x*	*y*	*z*	*U*_iso_*/*U*_eq_	
C1	0.40044 (15)	0.59196 (9)	0.16775 (9)	0.0177 (2)	
C2	0.38559 (16)	0.48697 (9)	0.14129 (9)	0.0192 (3)	
H2	0.3923	0.4590	0.0765	0.023*	
C3	0.35720 (15)	0.49975 (9)	0.29626 (9)	0.0160 (2)	
C4	0.40828 (17)	0.69554 (9)	0.33328 (9)	0.0213 (3)	
H4A	0.3204	0.7499	0.3025	0.032*	
H4B	0.5320	0.7228	0.3401	0.032*	
H4C	0.3892	0.6769	0.4010	0.032*	
C5	0.32784 (15)	0.46335 (9)	0.39394 (9)	0.0176 (2)	
H5	0.3457	0.3906	0.4109	0.021*	
C6	0.18665 (15)	0.54029 (9)	0.60895 (9)	0.0185 (2)	
H6A	0.0718	0.5716	0.5697	0.022*	
H6B	0.2737	0.5984	0.6330	0.022*	
C7	0.15188 (14)	0.47943 (9)	0.69873 (9)	0.0175 (2)	
C8	0.10500 (15)	0.37210 (9)	0.69078 (9)	0.0201 (3)	
H8	0.1018	0.3354	0.6286	0.024*	
C9	0.06311 (16)	0.31892 (10)	0.77312 (10)	0.0238 (3)	
H9	0.0328	0.2459	0.7684	0.029*	
C10	0.06662 (16)	0.37454 (11)	0.86169 (10)	0.0249 (3)	
C11	0.11210 (17)	0.48020 (11)	0.87283 (9)	0.0250 (3)	
H11	0.1132	0.5165	0.9350	0.030*	
C12	0.15620 (16)	0.53179 (10)	0.79027 (9)	0.0210 (3)	
H12	0.1900	0.6043	0.7965	0.025*	
N1	0.36001 (13)	0.42969 (8)	0.22186 (7)	0.0187 (2)	
N2	0.38326 (12)	0.60100 (7)	0.26758 (7)	0.0160 (2)	
N3	0.27904 (12)	0.52518 (8)	0.45773 (7)	0.0180 (2)	
N4	0.42710 (14)	0.67827 (8)	0.10515 (8)	0.0225 (2)	
O1	0.26029 (11)	0.46915 (6)	0.54488 (6)	0.01878 (19)	
O2	0.44180 (14)	0.76801 (7)	0.14148 (7)	0.0315 (2)	
O3	0.43216 (14)	0.65795 (8)	0.01587 (7)	0.0338 (2)	
F1	0.02098 (11)	0.32309 (7)	0.94206 (6)	0.0378 (2)	

### (E)-1-Methyl-5-nitro-1H-imidazole-2-carbaldehyde O-(4-fluorobenzyl) oxime (II) Atomic displacement parameters (Å^2^)

**Table:**

	*U*^11^	*U*^22^	*U*^33^	*U*^12^	*U*^13^	*U*^23^
C1	0.0181 (5)	0.0188 (6)	0.0166 (6)	0.0022 (4)	0.0048 (4)	0.0022 (5)
C2	0.0219 (6)	0.0197 (6)	0.0162 (6)	0.0018 (4)	0.0051 (4)	−0.0009 (5)
C3	0.0138 (5)	0.0155 (5)	0.0181 (6)	0.0007 (4)	0.0022 (4)	0.0002 (5)
C4	0.0275 (6)	0.0157 (6)	0.0215 (6)	−0.0017 (4)	0.0071 (5)	−0.0037 (5)
C5	0.0177 (5)	0.0164 (6)	0.0184 (6)	−0.0011 (4)	0.0030 (4)	0.0005 (5)
C6	0.0188 (5)	0.0187 (6)	0.0185 (6)	0.0011 (4)	0.0055 (4)	−0.0031 (5)
C7	0.0121 (5)	0.0208 (6)	0.0189 (6)	0.0023 (4)	0.0022 (4)	0.0004 (5)
C8	0.0180 (5)	0.0211 (6)	0.0206 (6)	0.0016 (4)	0.0031 (4)	−0.0007 (5)
C9	0.0214 (6)	0.0211 (6)	0.0286 (7)	0.0017 (5)	0.0049 (5)	0.0053 (5)
C10	0.0212 (6)	0.0328 (7)	0.0217 (6)	0.0045 (5)	0.0067 (5)	0.0111 (5)
C11	0.0237 (6)	0.0342 (7)	0.0174 (6)	0.0031 (5)	0.0053 (5)	−0.0008 (5)
C12	0.0188 (6)	0.0221 (6)	0.0218 (6)	0.0010 (4)	0.0037 (4)	−0.0012 (5)
N1	0.0203 (5)	0.0175 (5)	0.0181 (5)	0.0003 (4)	0.0042 (4)	−0.0013 (4)
N2	0.0164 (4)	0.0146 (5)	0.0172 (5)	0.0012 (4)	0.0041 (4)	0.0005 (4)
N3	0.0170 (5)	0.0200 (5)	0.0167 (5)	−0.0001 (4)	0.0032 (4)	0.0030 (4)
N4	0.0237 (5)	0.0212 (5)	0.0243 (6)	0.0027 (4)	0.0092 (4)	0.0038 (4)
O1	0.0230 (4)	0.0186 (4)	0.0164 (4)	0.0026 (3)	0.0077 (3)	0.0022 (3)
O2	0.0468 (6)	0.0176 (5)	0.0332 (6)	−0.0032 (4)	0.0158 (4)	0.0013 (4)
O3	0.0537 (6)	0.0307 (5)	0.0217 (5)	0.0033 (4)	0.0182 (4)	0.0037 (4)
F1	0.0437 (5)	0.0446 (5)	0.0286 (4)	0.0027 (4)	0.0158 (4)	0.0162 (4)

### (E)-1-Methyl-5-nitro-1H-imidazole-2-carbaldehyde O-(4-fluorobenzyl) oxime (II) Geometric parameters (Å, º)

**Table:**

C1—C2	1.3721 (16)	C6—H6A	0.9900
C1—N2	1.3789 (15)	C6—H6B	0.9900
C1—N4	1.4187 (15)	C7—C12	1.3890 (16)
C2—N1	1.3506 (15)	C7—C8	1.4005 (17)
C2—H2	0.9500	C8—C9	1.3890 (17)
C3—N1	1.3382 (15)	C8—H8	0.9500
C3—N2	1.3636 (15)	C9—C10	1.3757 (18)
C3—C5	1.4522 (16)	C9—H9	0.9500
C4—N2	1.4721 (15)	C10—F1	1.3679 (14)
C4—H4A	0.9800	C10—C11	1.3791 (19)
C4—H4B	0.9800	C11—C12	1.3888 (17)
C4—H4C	0.9800	C11—H11	0.9500
C5—N3	1.2731 (15)	C12—H12	0.9500
C5—H5	0.9500	N3—O1	1.4012 (12)
C6—O1	1.4393 (13)	N4—O2	1.2301 (14)
C6—C7	1.5013 (16)	N4—O3	1.2339 (14)
			
C2—C1—N2	108.14 (10)	C8—C7—C6	121.49 (11)
C2—C1—N4	127.25 (11)	C9—C8—C7	120.48 (11)
N2—C1—N4	124.60 (10)	C9—C8—H8	119.8
N1—C2—C1	109.26 (10)	C7—C8—H8	119.8
N1—C2—H2	125.4	C10—C9—C8	118.41 (12)
C1—C2—H2	125.4	C10—C9—H9	120.8
N1—C3—N2	112.56 (10)	C8—C9—H9	120.8
N1—C3—C5	119.63 (10)	F1—C10—C9	118.57 (12)
N2—C3—C5	127.81 (10)	F1—C10—C11	118.42 (12)
N2—C4—H4A	109.5	C9—C10—C11	123.01 (12)
N2—C4—H4B	109.5	C10—C11—C12	117.84 (12)
H4A—C4—H4B	109.5	C10—C11—H11	121.1
N2—C4—H4C	109.5	C12—C11—H11	121.1
H4A—C4—H4C	109.5	C11—C12—C7	121.26 (11)
H4B—C4—H4C	109.5	C11—C12—H12	119.4
N3—C5—C3	122.53 (11)	C7—C12—H12	119.4
N3—C5—H5	118.7	C3—N1—C2	105.71 (10)
C3—C5—H5	118.7	C3—N2—C1	104.31 (9)
O1—C6—C7	108.62 (9)	C3—N2—C4	126.92 (10)
O1—C6—H6A	110.0	C1—N2—C4	128.42 (10)
C7—C6—H6A	110.0	C5—N3—O1	110.43 (9)
O1—C6—H6B	110.0	O2—N4—O3	123.85 (11)
C7—C6—H6B	110.0	O2—N4—C1	119.16 (10)
H6A—C6—H6B	108.3	O3—N4—C1	116.99 (10)
C12—C7—C8	118.98 (11)	N3—O1—C6	107.89 (8)
C12—C7—C6	119.43 (10)		
			
N2—C1—C2—N1	−0.16 (13)	C5—C3—N1—C2	178.73 (10)
N4—C1—C2—N1	−179.62 (11)	C1—C2—N1—C3	0.72 (13)
N1—C3—C5—N3	−162.10 (10)	N1—C3—N2—C1	0.95 (12)
N2—C3—C5—N3	17.64 (18)	C5—C3—N2—C1	−178.81 (11)
O1—C6—C7—C12	152.70 (10)	N1—C3—N2—C4	−172.71 (10)
O1—C6—C7—C8	−30.95 (14)	C5—C3—N2—C4	7.53 (18)
C12—C7—C8—C9	0.13 (16)	C2—C1—N2—C3	−0.46 (12)
C6—C7—C8—C9	−176.23 (10)	N4—C1—N2—C3	179.02 (10)
C7—C8—C9—C10	0.82 (17)	C2—C1—N2—C4	173.07 (10)
C8—C9—C10—F1	178.37 (10)	N4—C1—N2—C4	−7.45 (18)
C8—C9—C10—C11	−0.87 (18)	C3—C5—N3—O1	179.79 (9)
F1—C10—C11—C12	−179.30 (10)	C2—C1—N4—O2	−178.34 (11)
C9—C10—C11—C12	−0.05 (18)	N2—C1—N4—O2	2.29 (17)
C10—C11—C12—C7	1.06 (18)	C2—C1—N4—O3	2.41 (18)
C8—C7—C12—C11	−1.10 (17)	N2—C1—N4—O3	−176.96 (11)
C6—C7—C12—C11	175.34 (10)	C5—N3—O1—C6	−173.96 (9)
N2—C3—N1—C2	−1.05 (13)	C7—C6—O1—N3	175.61 (8)

### (E)-1-Methyl-5-nitro-1H-imidazole-2-carbaldehyde O-(4-fluorobenzyl) oxime (II) Hydrogen-bond geometry (Å, º)

**Table:**

*D*—H···*A*	*D*—H	H···*A*	*D*···*A*	*D*—H···*A*
C4—H4*C*···N3	0.98	2.29	3.0184 (15)	131
C4—H4*A*···N1^i^	0.98	2.63	3.5693 (16)	160
C9—H9···N1^ii^	0.95	2.58	3.4973 (16)	163
C2—H2···O3^iii^	0.95	2.49	3.3165 (15)	145
C5—H5···O2^iv^	0.95	2.63	3.1676 (14)	116
C6—H6*A*···O2^v^	0.99	2.54	3.1376 (14)	119
C4—H4*C*···F1^vi^	0.98	2.77	3.353 (2)	119

Symmetry codes: (i) −*x*+1/2, *y*+1/2, −*z*+1/2; (ii) *x*−1/2, −*y*+1/2, *z*+1/2; (iii) −*x*+1, −*y*+1, −*z*; (iv) −*x*+1/2, *y*−1/2, −*z*+1/2; (v) *x*−1/2, −*y*+3/2, *z*+1/2; (vi) −*x*+1/2, *y*+1/2, −*z*+3/2.

### (E)-1-Methyl-5-nitro-1H-imidazole-2-carbaldehyde O-(4-bromobenzyl) oxime (III) Crystal data

**Table:**

C_12_H_11_BrN_4_O_3_	*F*(000) = 680
*M**_r_* = 339.16	*D*_x_ = 1.730 Mg m^−^^3^
Monoclinic, *P*2_1_/*n*	Mo *K*α radiation, λ = 0.71073 Å
*a* = 7.6024 (2) Å	Cell parameters from 6837 reflections
*b* = 12.7526 (3) Å	θ = 2.2–27.5°
*c* = 13.8954 (5) Å	µ = 3.17 mm^−^^1^
β = 104.869 (2)°	*T* = 120 K
*V* = 1302.05 (7) Å^3^	Block, yellow
*Z* = 4	0.66 × 0.52 × 0.24 mm

### (E)-1-Methyl-5-nitro-1H-imidazole-2-carbaldehyde O-(4-bromobenzyl) oxime (III) Data collection

**Table:**

Rigaku Mercury CCD diffractometer	2835 reflections with *I* > 2σ(*I*)
ω scans	*R*_int_ = 0.065
Absorption correction: multi-scan (*FS_ABSCOR*; Rigaku, 2013)	θ_max_ = 27.5°, θ_min_ = 2.2°
*T*_min_ = 0.438, *T*_max_ = 1.000	*h* = −8→9
16791 measured reflections	*k* = −16→16
2974 independent reflections	*l* = −18→16

### (E)-1-Methyl-5-nitro-1H-imidazole-2-carbaldehyde O-(4-bromobenzyl) oxime (III) Refinement

**Table:**

Refinement on *F*^2^	Primary atom site location: structure-invariant direct methods
Least-squares matrix: full	Hydrogen site location: inferred from neighbouring sites
*R*[*F*^2^ > 2σ(*F*^2^)] = 0.051	H-atom parameters constrained
*wR*(*F*^2^) = 0.140	*w* = 1/[σ^2^(*F*_o_^2^) + (0.0837*P*)^2^ + 1.3179*P*] where *P* = (*F*_o_^2^ + 2*F*_c_^2^)/3
*S* = 1.11	(Δ/σ)_max_ = 0.001
2974 reflections	Δρ_max_ = 1.78 e Å^−^^3^
182 parameters	Δρ_min_ = −1.03 e Å^−^^3^
0 restraints	

### (E)-1-Methyl-5-nitro-1H-imidazole-2-carbaldehyde O-(4-bromobenzyl) oxime (III) Special details

**Table:**

Geometry. All esds (except the esd in the dihedral angle between two l.s. planes) are estimated using the full covariance matrix. The cell esds are taken into account individually in the estimation of esds in distances, angles and torsion angles; correlations between esds in cell parameters are only used when they are defined by crystal symmetry. An approximate (isotropic) treatment of cell esds is used for estimating esds involving l.s. planes.

### (E)-1-Methyl-5-nitro-1H-imidazole-2-carbaldehyde O-(4-bromobenzyl) oxime (III) Fractional atomic coordinates and isotropic or equivalent isotropic displacement parameters (Å^2^)

**Table:**

	*x*	*y*	*z*	*U*_iso_*/*U*_eq_	
C1	0.4010 (4)	0.5860 (2)	0.1628 (2)	0.0210 (6)	
C2	0.3825 (4)	0.4812 (2)	0.1385 (2)	0.0227 (6)	
H2	0.3902	0.4517	0.0769	0.027*	
C3	0.3498 (4)	0.4984 (2)	0.2868 (2)	0.0201 (6)	
C4	0.3974 (5)	0.6933 (2)	0.3184 (3)	0.0281 (7)	
H4A	0.3108	0.7457	0.2827	0.042*	
H4B	0.5214	0.7210	0.3308	0.042*	
H4C	0.3704	0.6770	0.3821	0.042*	
C5	0.3150 (4)	0.4652 (2)	0.3803 (2)	0.0221 (6)	
H5	0.3120	0.3922	0.3932	0.026*	
C6	0.2011 (4)	0.5460 (2)	0.5916 (2)	0.0227 (6)	
H6A	0.0894	0.5833	0.5554	0.027*	
H6B	0.2982	0.5986	0.6159	0.027*	
C7	0.1655 (4)	0.4863 (2)	0.6778 (2)	0.0203 (6)	
C8	0.1233 (4)	0.3798 (2)	0.6718 (2)	0.0229 (6)	
H8	0.1207	0.3422	0.6124	0.027*	
C9	0.0849 (4)	0.3284 (2)	0.7522 (2)	0.0237 (6)	
H9	0.0561	0.2558	0.7481	0.028*	
C10	0.0890 (4)	0.3840 (2)	0.8382 (2)	0.0224 (6)	
C11	0.1292 (4)	0.4900 (2)	0.8463 (2)	0.0232 (6)	
H11	0.1300	0.5275	0.9055	0.028*	
C12	0.1683 (4)	0.5400 (2)	0.7656 (2)	0.0232 (6)	
H12	0.1977	0.6126	0.7702	0.028*	
N1	0.3517 (4)	0.42704 (18)	0.21619 (19)	0.0233 (5)	
N2	0.3819 (3)	0.59752 (17)	0.25814 (18)	0.0191 (5)	
N3	0.2882 (3)	0.52867 (18)	0.44583 (19)	0.0219 (5)	
N4	0.4338 (4)	0.6687 (2)	0.1020 (2)	0.0234 (5)	
O1	0.2561 (3)	0.47297 (15)	0.52654 (16)	0.0239 (5)	
O2	0.4605 (4)	0.75804 (16)	0.13734 (19)	0.0325 (5)	
O3	0.4324 (4)	0.64727 (19)	0.01529 (19)	0.0343 (6)	
Br1	0.04121 (4)	0.31299 (2)	0.94938 (2)	0.02821 (16)	

### (E)-1-Methyl-5-nitro-1H-imidazole-2-carbaldehyde O-(4-bromobenzyl) oxime (III) Atomic displacement parameters (Å^2^)

**Table:**

	*U*^11^	*U*^22^	*U*^33^	*U*^12^	*U*^13^	*U*^23^
C1	0.0257 (14)	0.0171 (12)	0.0208 (14)	0.0008 (10)	0.0071 (11)	−0.0021 (10)
C2	0.0304 (15)	0.0168 (12)	0.0214 (15)	0.0020 (10)	0.0076 (12)	−0.0035 (10)
C3	0.0216 (13)	0.0147 (11)	0.0239 (15)	0.0002 (10)	0.0058 (11)	−0.0007 (10)
C4	0.0431 (19)	0.0137 (13)	0.0295 (18)	−0.0020 (11)	0.0132 (15)	−0.0045 (10)
C5	0.0241 (14)	0.0144 (11)	0.0284 (16)	0.0010 (10)	0.0078 (12)	0.0019 (10)
C6	0.0284 (15)	0.0165 (12)	0.0237 (15)	0.0008 (10)	0.0079 (12)	0.0004 (10)
C7	0.0198 (13)	0.0173 (12)	0.0231 (15)	0.0017 (10)	0.0042 (11)	0.0025 (10)
C8	0.0259 (14)	0.0168 (12)	0.0256 (15)	0.0013 (10)	0.0058 (11)	−0.0016 (10)
C9	0.0279 (15)	0.0140 (11)	0.0291 (17)	0.0004 (10)	0.0072 (12)	0.0008 (11)
C10	0.0218 (14)	0.0196 (13)	0.0262 (15)	0.0019 (10)	0.0069 (11)	0.0052 (10)
C11	0.0275 (15)	0.0202 (13)	0.0221 (15)	0.0010 (11)	0.0067 (12)	−0.0016 (10)
C12	0.0290 (15)	0.0141 (12)	0.0269 (16)	−0.0015 (10)	0.0082 (12)	0.0000 (10)
N1	0.0305 (13)	0.0144 (10)	0.0255 (13)	−0.0002 (9)	0.0083 (10)	−0.0035 (9)
N2	0.0261 (12)	0.0126 (10)	0.0195 (12)	−0.0001 (8)	0.0071 (9)	−0.0015 (9)
N3	0.0263 (12)	0.0176 (10)	0.0227 (13)	−0.0007 (9)	0.0079 (10)	0.0041 (9)
N4	0.0268 (13)	0.0188 (11)	0.0257 (14)	0.0005 (9)	0.0090 (11)	0.0011 (10)
O1	0.0328 (12)	0.0170 (9)	0.0253 (12)	0.0019 (8)	0.0137 (9)	0.0027 (8)
O2	0.0485 (14)	0.0161 (10)	0.0357 (13)	−0.0055 (9)	0.0158 (11)	−0.0015 (9)
O3	0.0536 (15)	0.0290 (12)	0.0251 (13)	0.0010 (11)	0.0190 (11)	−0.0004 (10)
Br1	0.0366 (2)	0.0231 (2)	0.0267 (2)	0.00051 (10)	0.01133 (17)	0.00691 (10)

### (E)-1-Methyl-5-nitro-1H-imidazole-2-carbaldehyde O-(4-bromobenzyl) oxime (III) Geometric parameters (Å, º)

**Table:**

C1—C2	1.377 (4)	C6—H6A	0.9900
C1—N2	1.378 (4)	C6—H6B	0.9900
C1—N4	1.413 (4)	C7—C8	1.393 (4)
C2—N1	1.352 (4)	C7—C12	1.394 (4)
C2—H2	0.9500	C8—C9	1.389 (4)
C3—N1	1.341 (4)	C8—H8	0.9500
C3—N2	1.366 (3)	C9—C10	1.383 (4)
C3—C5	1.453 (4)	C9—H9	0.9500
C4—N2	1.468 (3)	C10—C11	1.384 (4)
C4—H4A	0.9800	C10—Br1	1.905 (3)
C4—H4B	0.9800	C11—C12	1.387 (4)
C4—H4C	0.9800	C11—H11	0.9500
C5—N3	1.273 (4)	C12—H12	0.9500
C5—H5	0.9500	N3—O1	1.401 (3)
C6—O1	1.433 (4)	N4—O3	1.232 (4)
C6—C7	1.502 (4)	N4—O2	1.237 (3)
			
C2—C1—N2	108.0 (3)	C12—C7—C6	118.9 (2)
C2—C1—N4	126.9 (3)	C9—C8—C7	120.3 (3)
N2—C1—N4	125.1 (2)	C9—C8—H8	119.8
N1—C2—C1	109.1 (3)	C7—C8—H8	119.8
N1—C2—H2	125.4	C10—C9—C8	119.4 (3)
C1—C2—H2	125.4	C10—C9—H9	120.3
N1—C3—N2	112.2 (3)	C8—C9—H9	120.3
N1—C3—C5	119.7 (2)	C9—C10—C11	121.8 (3)
N2—C3—C5	128.1 (3)	C9—C10—Br1	119.4 (2)
N2—C4—H4A	109.5	C11—C10—Br1	118.8 (2)
N2—C4—H4B	109.5	C10—C11—C12	118.1 (3)
H4A—C4—H4B	109.5	C10—C11—H11	120.9
N2—C4—H4C	109.5	C12—C11—H11	120.9
H4A—C4—H4C	109.5	C11—C12—C7	121.5 (3)
H4B—C4—H4C	109.5	C11—C12—H12	119.2
N3—C5—C3	123.6 (3)	C7—C12—H12	119.2
N3—C5—H5	118.2	C3—N1—C2	105.9 (2)
C3—C5—H5	118.2	C3—N2—C1	104.7 (2)
O1—C6—C7	108.3 (2)	C3—N2—C4	126.7 (3)
O1—C6—H6A	110.0	C1—N2—C4	128.6 (2)
C7—C6—H6A	110.0	C5—N3—O1	110.1 (2)
O1—C6—H6B	110.0	O3—N4—O2	123.5 (3)
C7—C6—H6B	110.0	O3—N4—C1	117.4 (2)
H6A—C6—H6B	108.4	O2—N4—C1	119.1 (3)
C8—C7—C12	118.9 (3)	N3—O1—C6	108.3 (2)
C8—C7—C6	122.2 (3)		
			
N2—C1—C2—N1	0.2 (3)	C5—C3—N1—C2	178.5 (3)
N4—C1—C2—N1	−179.8 (3)	C1—C2—N1—C3	0.5 (3)
N1—C3—C5—N3	−170.9 (3)	N1—C3—N2—C1	1.3 (3)
N2—C3—C5—N3	8.7 (5)	C5—C3—N2—C1	−178.3 (3)
O1—C6—C7—C8	−23.1 (4)	N1—C3—N2—C4	−177.1 (3)
O1—C6—C7—C12	159.4 (3)	C5—C3—N2—C4	3.3 (5)
C12—C7—C8—C9	−0.1 (4)	C2—C1—N2—C3	−0.9 (3)
C6—C7—C8—C9	−177.7 (3)	N4—C1—N2—C3	179.2 (3)
C7—C8—C9—C10	0.0 (5)	C2—C1—N2—C4	177.4 (3)
C8—C9—C10—C11	0.5 (5)	N4—C1—N2—C4	−2.5 (5)
C8—C9—C10—Br1	−178.8 (2)	C3—C5—N3—O1	179.2 (2)
C9—C10—C11—C12	−0.9 (4)	C2—C1—N4—O3	6.0 (5)
Br1—C10—C11—C12	178.4 (2)	N2—C1—N4—O3	−174.0 (3)
C10—C11—C12—C7	0.8 (4)	C2—C1—N4—O2	−174.7 (3)
C8—C7—C12—C11	−0.3 (4)	N2—C1—N4—O2	5.3 (4)
C6—C7—C12—C11	177.3 (3)	C5—N3—O1—C6	−171.8 (2)
N2—C3—N1—C2	−1.2 (3)	C7—C6—O1—N3	179.7 (2)

### (E)-1-Methyl-5-nitro-1H-imidazole-2-carbaldehyde O-(4-bromobenzyl) oxime (III) Hydrogen-bond geometry (Å, º)

**Table:**

*D*—H···*A*	*D*—H	H···*A*	*D*···*A*	*D*—H···*A*
C4—H4*C*···N3	0.98	2.24	2.997 (4)	133
C4—H4*A*···N1^i^	0.98	2.62	3.499 (4)	149
C9—H9···N1^ii^	0.95	2.77	3.681 (4)	160
C2—H2···O3^iii^	0.95	2.44	3.282 (4)	148
C5—H5···O2^iv^	0.95	2.64	3.341 (4)	131
C6—H6*A*···O2^v^	0.99	2.63	3.254 (4)	121
C4—H4*C*···Br1^vi^	0.98	2.85	3.491 (3)	124

Symmetry codes: (i) −*x*+1/2, *y*+1/2, −*z*+1/2; (ii) *x*−1/2, −*y*+1/2, *z*+1/2; (iii) −*x*+1, −*y*+1, −*z*; (iv) −*x*+1/2, *y*−1/2, −*z*+1/2; (v) *x*−1/2, −*y*+3/2, *z*+1/2; (vi) −*x*+1/2, *y*+1/2, −*z*+3/2.
