# Supplementary material for: Different mol­ecular conformations in the crystal structures of three 5-nitro­imidazolyl derivatives
Source: Acta Crystallogr E Crystallogr Commun. 2018 Feb 23;74(Pt 3):380–4. doi: 10.1107/S2056989018002876 (PMC5947808; doi:10.1107/S2056989018002876)
Supplement: Supplementary file 8 [file e-74-00380-sup8.pdf]

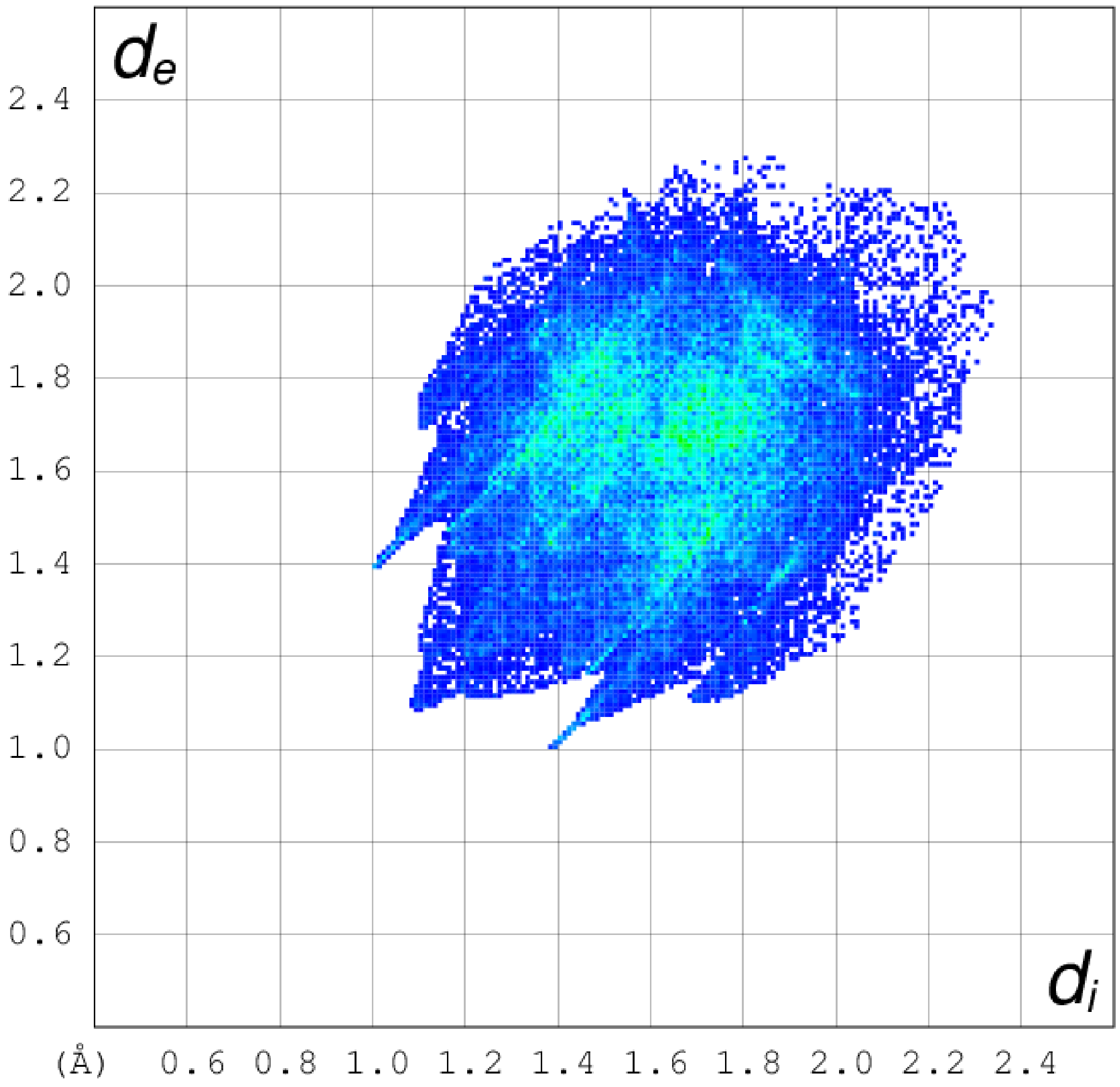

Hirshfeld fingerprint plot for (I), $C_{12}H_{12}N_4O_3$

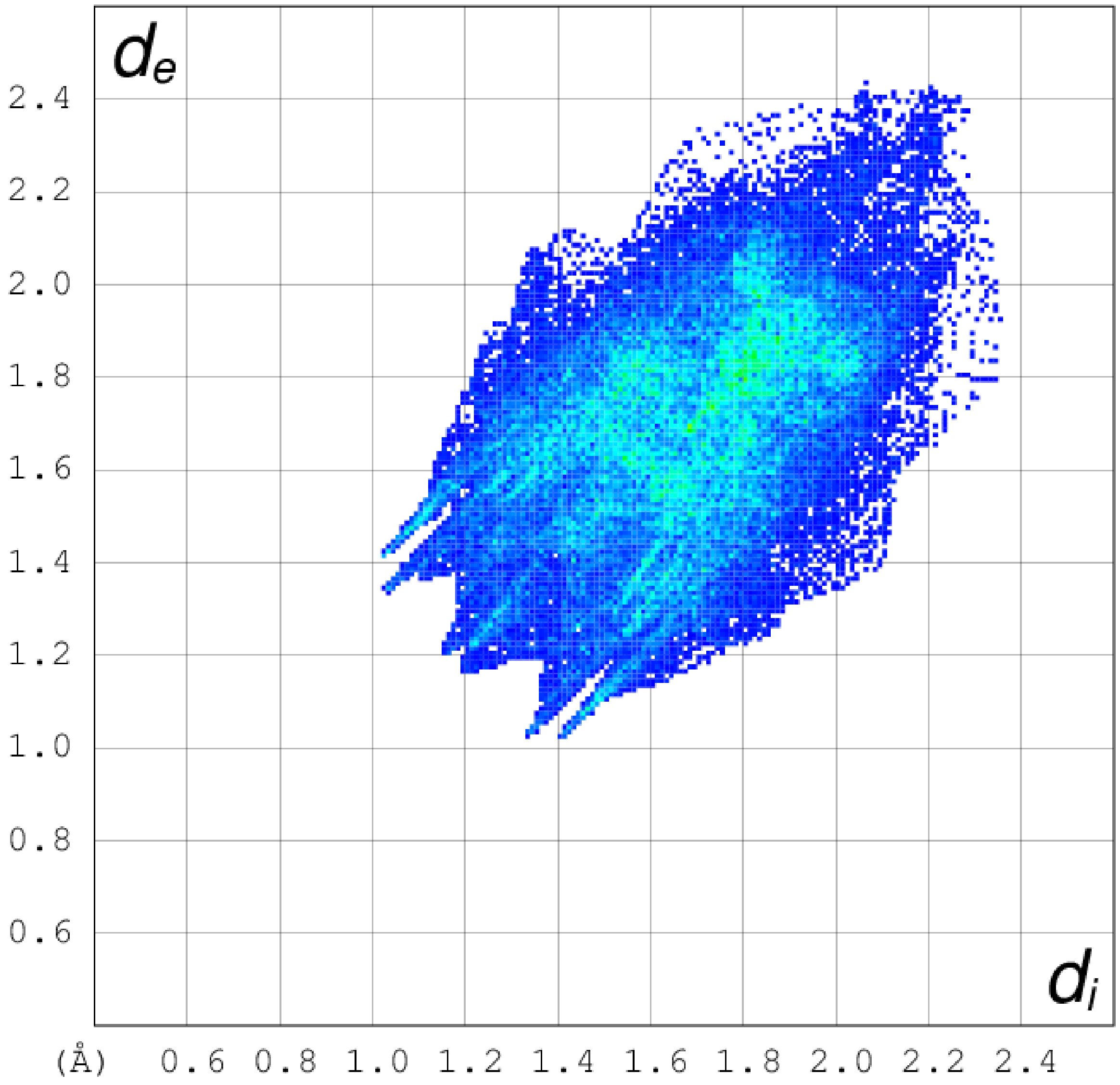

Hirshfeld fingerprint plot for (II), $C_{12}H_{11}FN_4O_3$

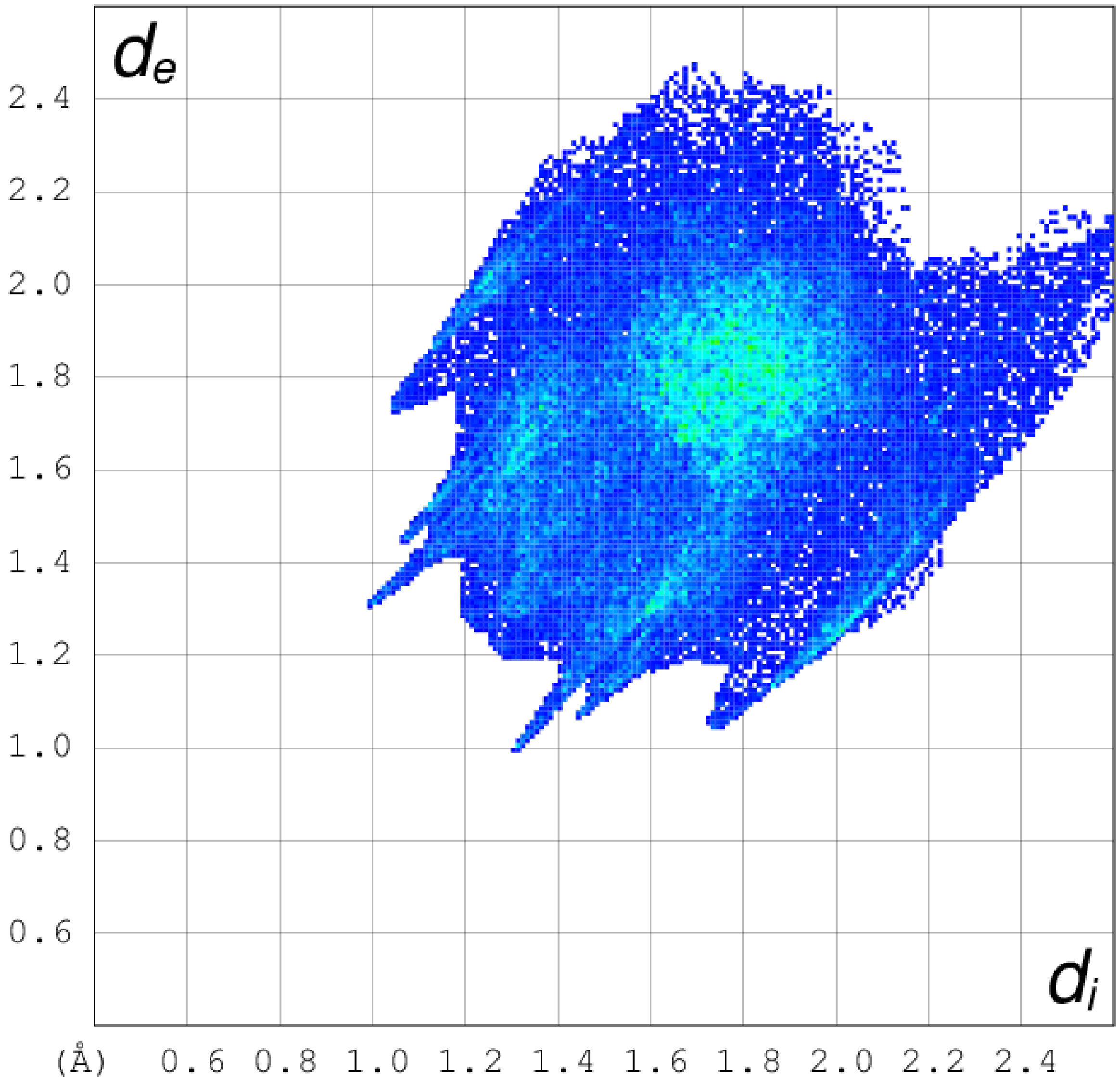

Hirshfeld fingerprint plot for (III), $C_{12}H_{11}BrN_4O_3$
